# Case Report: Endoluminal removal of a conical retrievable superior vena cava filter with a retraction hook attached to the wall

**DOI:** 10.3389/fcvm.2024.1412571

**Published:** 2024-07-02

**Authors:** Xuan Tian, Jianlong Liu, Jinyong Li, Xiao Liu, Mi Zhou, Yule Tian

**Affiliations:** ^1^ Department of Vascular Surgery, Beijing Jishuitan Hospital, Capital Medical University, Beijing, China; ^2^ International Department, Experimental High School, Beijing Normal University, Beijing, China

**Keywords:** superior vena cava filter, filter retrieval, case report, loop-snare, superior vena cava (SVC)

## Abstract

We report the case of a 22-year-old male who underwent endoluminal surgery and was implanted an Option Elite filter in the superior vena cava (SVC) while the filter retraction hook was attached to the vessel wall. The patient requested to remove the filter after 155 days. Preoperative ultrasonography and CT examination revealed that the filter retraction hook was very likely to penetrate the SVC wall and its tip was very close to the right pulmonary artery. The SVC was not obstructed, and no thrombus was observed in either upper limb. After the filter retrieval device (ZYLOX, China) failed to capture the filter hook, we introduced a pigtail catheter with its tip partly removed and a loach guidewire, used a modified loop-snare technique to cut the proliferative tissues and free the hook, and finally removed the filter successfully by direct suspension of the guidewire. During this procedure, the patient experienced discomfort, such as chest pain and palpitations, but these symptoms disappeared when procedure completed. Repeated multiangle angiography revealed no contrast medium extravasation, no complications such as pericardial tamponade, pleural effusion, SVC haematoma formation, right pulmonary artery dissecting aneurysm, or intramural haematoma. We initially presented the modified loop-snare technique used to remove a conical superior vena cava filter (SVCF), so this method can be considered a practical and novel auxiliary technique for successful filter retrieval.

## Introduction

Vena cava filters are mainly used in patients with deep vein thrombosis (DVT) to prevent fatal pulmonary embolism (PE). The incidence of PE after DVT of lower extremities is as high as 45%–50% (1, 2), so inferior vena cava filters (IVCFs) are widely used. However, recent studies have shown that the incidence of PE after DVT in the upper limbs can reach 5%–10% (3). Some scholars have attempted to place the filter in the SVC, but this practice is controversial (4).

In view of the multiple complications associated with permanent filter implantation (5, 6), retrievable filters are dominant in trend, and timely removal is recommended once PE risk is reduced and the filter is no longer needed (7–9). At present, endovascular surgery is preferred to remove IVCFs. However, open surgery is essential when filter removal is failed due to serious complications and can't be treated via endoluminal approach (10). Filter tilt is defined as an angulation of more than 15 degrees from the filter's long axis, which occurs in 3%–9% cases (11), resulting in hook attached to the vessel wall with proliferative tissue wrapping, failure to capture, thus increased damage of the hook or strut perforation to the vascular wall and adjacent tissues, and at last failure to retrieve the filter. Severe tilt is more common with IVCFs, while reports of tilt with SVCFs are rare. However, severe inclinations may result in permanent filter implantation into the SVC (4).

In this study, we report a case of endoluminal removal of a conical superior vena cava retrievable filter with the hook attached to the vessel wall.

## Case report

The patient is a 22-year-old male who underwent the surgery to treat the thoracic outlet syndrome 155 days ago. Postoperative symptoms of left brachial plexus injury occurred with left upper limb DVT and PE. An Option Elite vena cava filter (ARGON, USA) was placed in the SVC, and anticoagulant therapy of 20 mg oral rivaroxaban QD was administered for 3 months. In the attempts to remove the SVCF, difficulties were encountered, and the filter could not be removed. The patient had repeated visits to two hospitals but still failed to remove the filter, so he was eventually transferred to our hospital for filter removal.

### Preoperative examination

**Figure 1:**
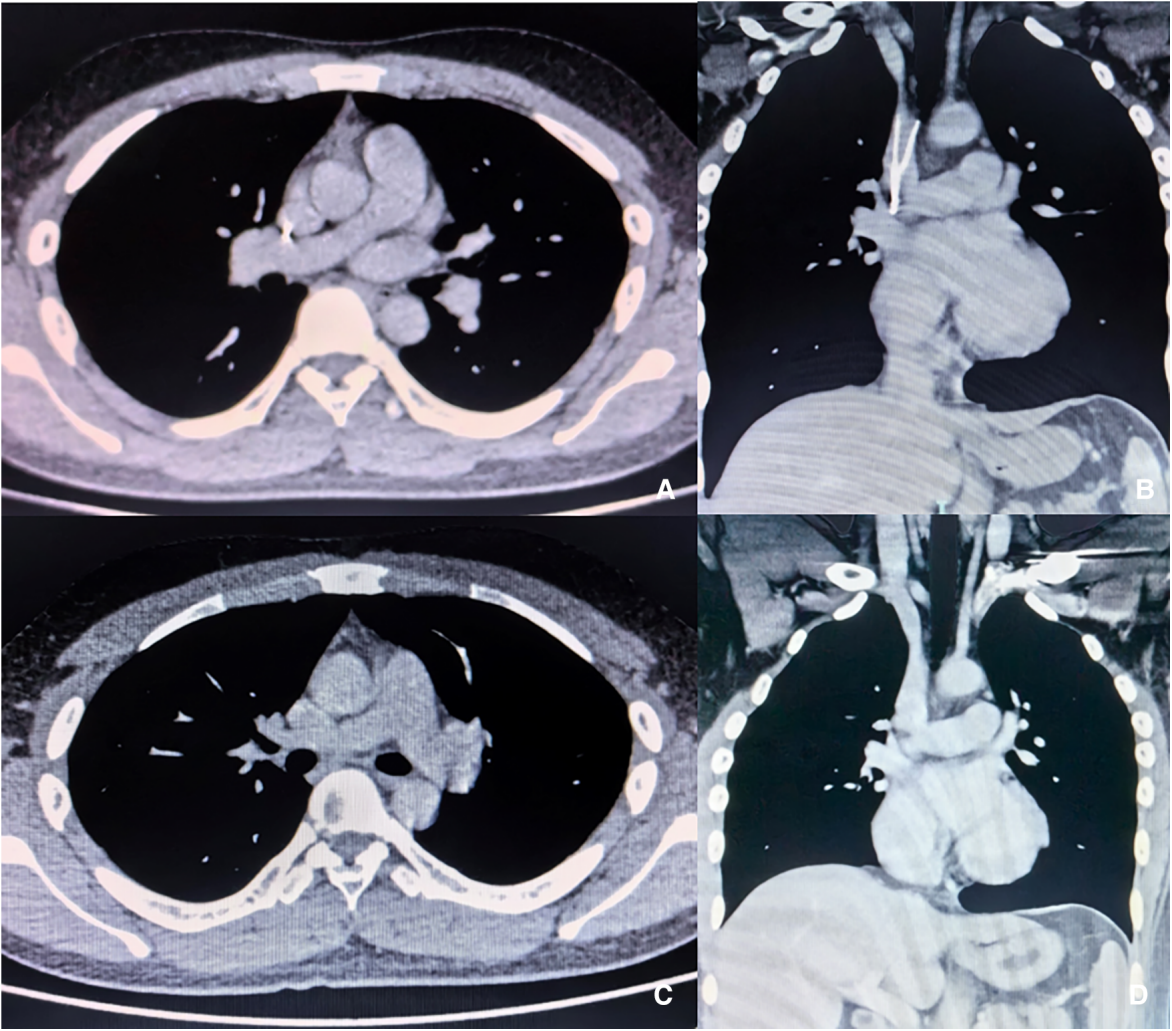
Enhanced CT images before and after procedure. (**A,B**) The filter retraction hook entered SVC wall, and the tip was close to the right pulmonary artery. (**C,D**) No haematoma, dissection or cardiac fluid accumulation in the superior vena cava or right pulmonary artery after filter removal.

No PE was detected via computed tomographic pulmonary angiography (CTPA), the SVC was patent, and no haematoma formation. The filter retraction hook was likely to penetrate the SVC wall, and its tip was very close to the right pulmonary artery (Figures 1A,B). Colour Doppler ultrasound revealed no thrombus in the deep veins of either upper limb. No pericardial effusion was observed in echocardiography, nor arrhythmia in electrocardiogram. The surgical indications for filter removal were met, and the patient had strong desire for filter removal surgery. The procedure plan was designed as follows: the modified loop-snare technique (12) was firstly attempted to remove the filter; open surgery was a back-up when endoluminal therapy failed or complications occurred.

### Device preparation

4Fr 100 cm pigtail catheter (Cordis, USA), a 4F catheter is recommended for its relative smaller outer diameter and easier advancement; 260 cm angled hydrophilic coated guidewire (Terumo Medical, Japan); filter retrieval kit (ZYLOX, China), a domestic manufactured device which is similar to Bard snare retrieval kit (BD, USA) in structure and function.

**Figure 2:**
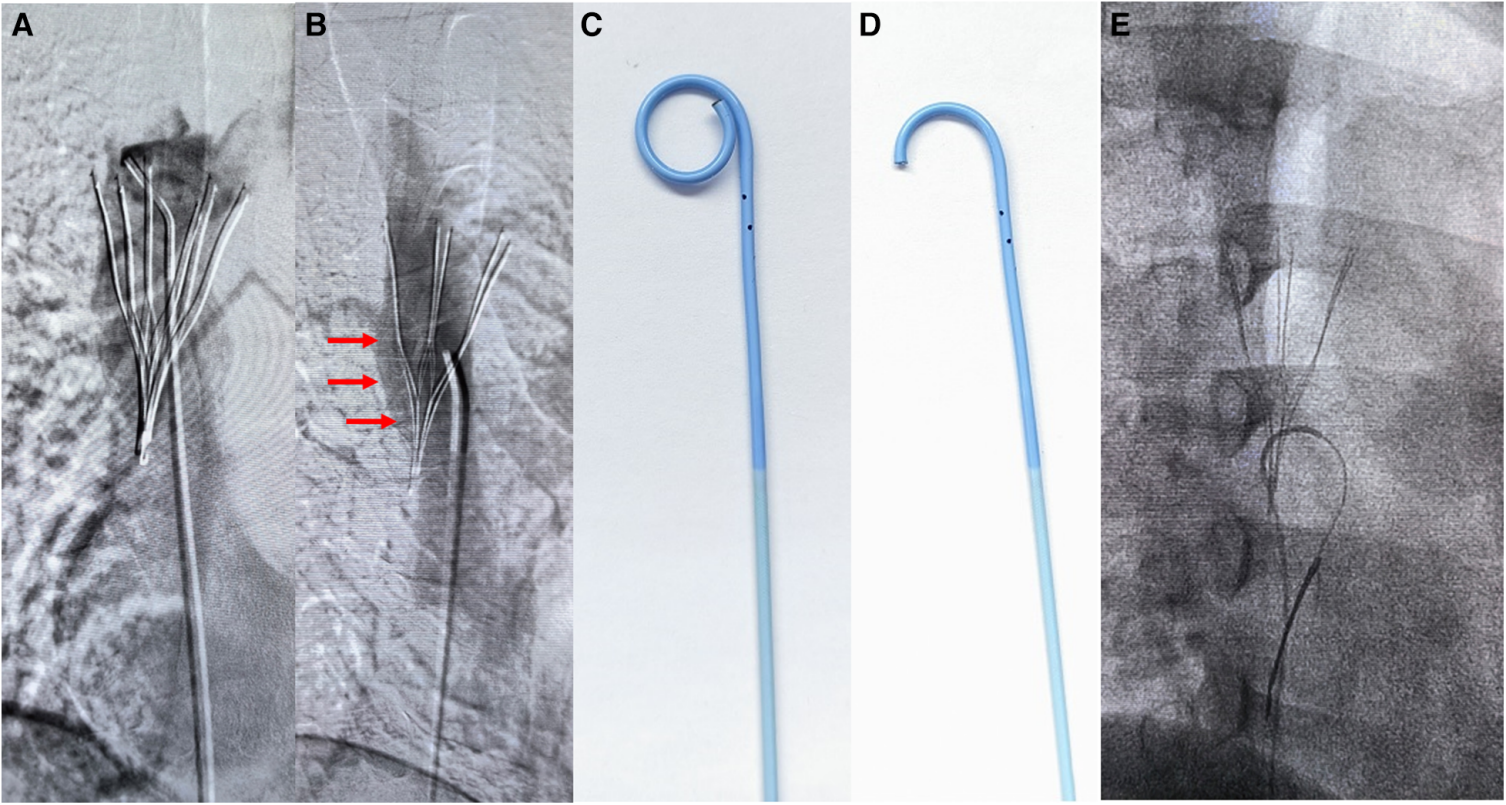
Images of the loop-snare technique steps. (**A,B**) Multiangle angiography of the superior vena cava. Red arrows marked the interspace between the filter and the SVC wall where the wire loop was formed. (**C,D**) The pigtail catheter shape with its tip partly cut. (**E**) Modified loop-snare technique using a decapitated pigtail catheter.

**Figure 3:**
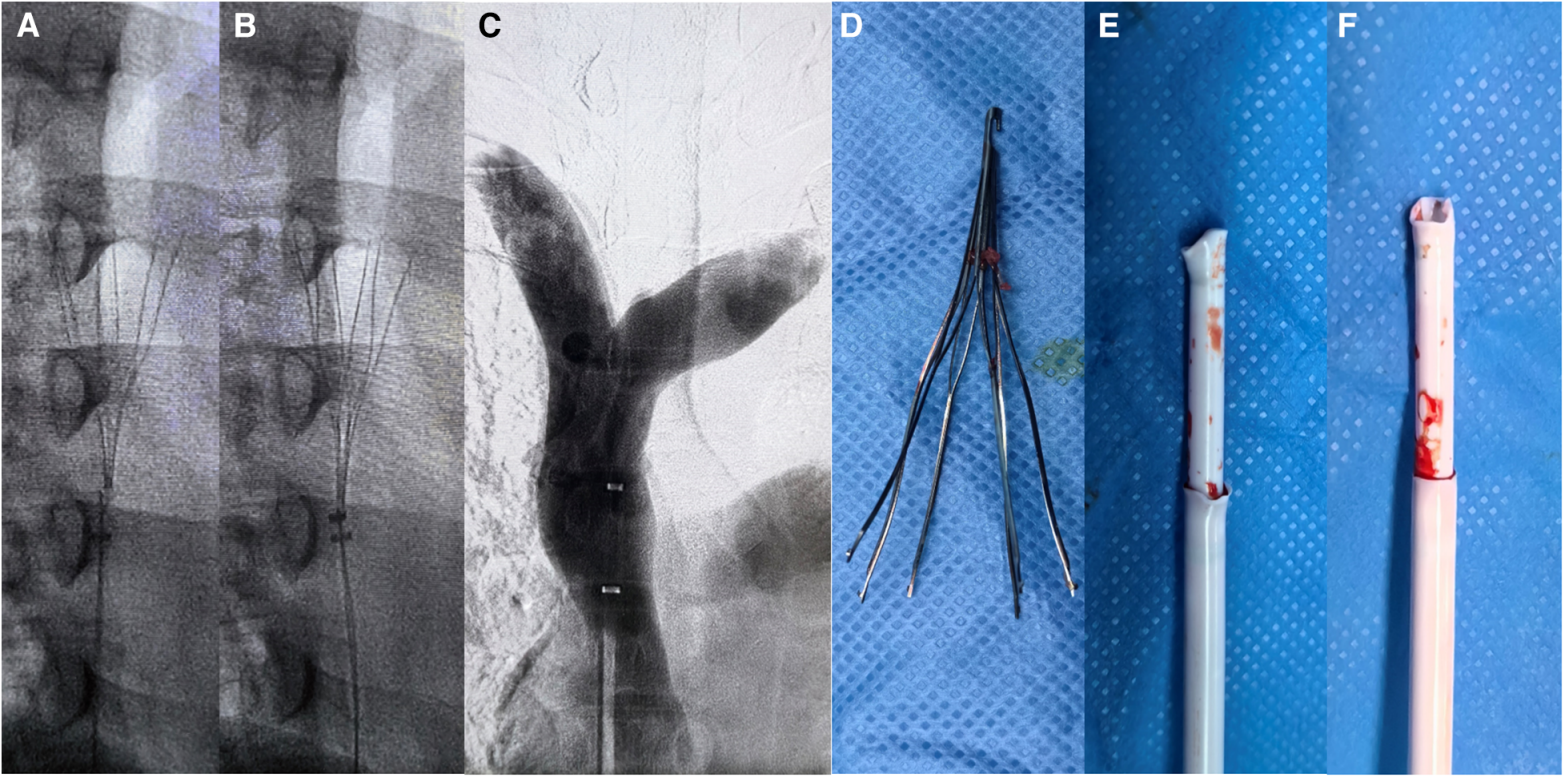
Filter removal process images. (**A,B**) Modified loop-snare technique for cutting the proliferated tissue around the retraction hook, with the guidewire directly suspending the retraction hook. (**C,D**) After the filter was successfully removed, no damage to the superior vena cava was observed by contrast imaging, and the filter was completely removed. (**E,F**) The deformed shape of the filter retrieval devices.

Angiographic imaging of the right common femoral vein showed that the inferior vena cava was unobstructed and no thrombus observed. Repeated multiangle angiography showed that the filter retraction hook had possibly entered the SVC wall (Figures 2A,B). ZYLOX filter retrieval kit was introduced but difficulties were encountered when attempting to capture the filter retraction hook. To solve this issue, we utilized a modified loop-snare technique (13) by introducing a pigtail catheter and a hydrophilic guidewire (Figure 2E). The pigtail catheter had its tip partially cut (Figures 2C,D) and was rotated to be guided into the interspace between the filter and the SVC wall (Figure 2B, red arrows). The guidewire was used to increase the catheter support and was advanced to free its end to be snared and externalized. A wire loop was formed across the proliferative tissue and was used to cut and destroy the proliferative tissue surrounding the retraction hook through the exertion of counteracting forces by the guidewire and retrieval sheath (Figures 3A,B), and the filter was successfully removed by directly suspension of the guidewire. It is worth noting that the patient experienced uncomfortable symptoms such as chest pain and palpitations during the removal process, but these symptoms disappeared once the operation was successfully completed. Repeated multiangle angiography showed no contrast medium spillage (Figure 3C). Finally, the filter was removed successfully and completely (Figure 3D). We also observed that the tip of the retrieval sheath was significantly deformed (Figures 3E,F).

CTPA (Figures 1C,D) was performed one week after the procedure. No pericardial effusion or pleural effusion was observed, no haematoma was found in the SVC, and no right pulmonary artery dissecting aneurysm or intramural haematoma was observed. PE did not occur in perioperative period, and anticoagulant therapy with 20 mg of rivaroxaban QD was recommended after the procedure (14). There was no recurrence of upper extremity DVT, no SVC thrombosis, and no symptoms related to PE during the 3-month follow-up.

## Discussion

Compared with the inferior vena cava, SVC is short in length and small in diameter (15), so it is relatively difficult to place the filter. In particular for conical filters, inaccurate positioning and severe tilt are more likely to occur (4). This may lead to complications such as pericardial effusion, arrhythmia, aortic dissection, arteriovenous fistula, haemothorax, pneumothorax and air embolism (16–18). Especially in the case of hook attached to the vessel wall, the filter cannot be retrieved successfully and lead to permanent implantation in the body (4).

The surgical procedure in this case has the following characteristics and challenges: (1) the filter retraction hook is embedded in the SVC wall, it may be difficult to be captured by the conventional retrieval snare device, and the filter tip is close to the right pulmonary artery, so there are risks of collateral damage; (2) the occurrence of bleeding may result in complications such as pericardial tamponade, arrhythmia and pleural effusion, so it is vital to avoid the vascular injury during the procedure; and (3) there are no reports on the use of the modified loop-snare technique to remove the attached SVCF.

When the filter is heavily tilt against the wall, the tissue proliferation around the retraction hook will result in the failure of capturing the retraction hook properly. The following methods can significantly increase the success rate of endoluminal filter removal: (1) loop-snare technique (19), which can pull the filter to correct its tilt angle; (2) the modified loop-snare technique (12), which is adopted in this study; (3) double wire lassoing technique (20), which corrects the tilt angle by pulling from both ends of the filter simultaneously; and (4) biopsy forceps technique (12), which grabs the filter retraction hook and struts into the catheter for filter removal.

We highlight the risk of central vein breakage during the removal of the conical filter with the retraction hook attached to the wall using various methods. When the inferior vena cava is injured, the bleeding risk is low due to the lower pressure of the central vein, and it's surrounded by the vascular sheath and adipose tissue. Once bleeding occurs, the haematoma can be absorbed by the posterior peritoneum quickly without major complications. However, if the injury occurs in the SVC, which is surrounded by the pericardium, the procedure risk increases significantly as the injury may cause pericardial effusion and other complications, such as hypotension and dyspnoea (18). When comparing the two loop-snare techniques, the modified loop-snare technique is safer to cut the proliferative tissue around the hook because it allows the filter retraction hook to re-enter the SVC. If the standard loop-snare technique is applied, pulling the filter may cause the hook to stab into the right pulmonary artery and cause serious complications. For this reason, the modified loop-snare technique is selected in this case.

Currently, the filter deployed in the superior vena cava is still controversial (4), but if the filter is needed, we recommend the following choices: (1) Denali filter (BD, USA), that it has good radial support in the vessel, and the risk of filter tilt with its hook attached to the wall is low (21); and (2) Non-conical filter, such as Optease (Cordis, USA), that it should be released in the direction of blood flow to avoid dislocation, and the filter should be retrieved at an early stage to avoid permanent placement; (3) Temperfilter II filter (B. Braun, Germany), the filter should be inverted via the femoral vein to the superior vena cava and should be avoided for long dwelling time.

## Conclusion

Conical SVCF retraction hook attachment is a rare complication that can lead to permanent indwelling of the filter. However, the filter can be successfully removed with the modified loop-snare technique without any complications, thus avoiding severe trauma associated with open surgery. This new practical and auxiliary technique provides a valid method for the removal of SVCFs and has academic value and practical application prospects.

## Data Availability

The original contributions presented in the study are included in the article/Supplementary Material, further inquiries can be directed to the corresponding author.

## References

[1] Nutescu EA Crivera C Schein JR Bookhart BK. Incidence of hospital readmission in patients diagnosed with DVT and PE: clinical burden of recurrent events. Int J Clin Pract. (2015) 69:321–7. 10.1111/ijcp.12519 25395271

[2] Tian X Liu JL Gu JP Xu H Ni FC Li Z A multicenter clinical trial of safety and effectiveness of octoparms® vena cava filter in preventing pulmonary embolism. Chin J Gen Surg. (2021) 30(12):1395–402. 10.7659/j.issn.1005-6947.2021.12.002

[3] Rokosh RS Ranganath N Yau P Rockman C Sadek M Berland T High prevalence and mortality associated with upper extremity deep venous thrombosis in hospitalized patients at a tertiary care center. Ann Vasc Surg. (2020) 65:55–65. 10.1016/j.avsg.2019.10.055 31669473

[4] Lopera JE Barnes L. A single center 10-year clinical experience with superior vena cava retrievable filters. Catheter Cardiovasc Interv. (2019) 95(1):1–6. 10.1002/ccd.28533 31609084

[5] Moriarty JM Steinberger JD Bansal AK. Inferior vena cava filters: when to place and when to remove. Semin Respir Crit Care Med. (2017) 38(1):84–93. 10.1055/s-0036-1597558 28208202

[6] Quencer KB Smith TA Deipolyi A Mojibian H Ayyagari R Latich I Procedural complications of inferior vena cava filter retrieval, an illustrated review. CVIR Endovasc. (2020) 3(1):23. 10.1186/s42155-020-00113-6 32337618 PMC7184068

[7] Streiff MB Agnelli G Connors JM Crowther M Eichinger S Lopes R Guidance for the treatment of deep vein thrombosis and pulmonary embolism. J Thromb Thrombolysis. (2016) 41(1):32–67. 10.1007/s11239-015-1317-0 26780738 PMC4715858

[8] Interventional physicians branch of Chinese medical doctor association. Expert consensuses on the codes for the insertion and removal of inferior vena cava filters (2nd edition). Nat Med J Chin. (2020) 100(27):2092–101. 10.3760/cma.j.cn112137-20200317-00804

[9] Vascular surgery group, Gurgery branch, Chinese medical association. The interpretation of clinical application guidelines of vena cava filter. Chin J Vasc Surg. (2019) 4(3):145–53. 10.3760/cma.j.issn.2096-1863.2019.03.005

[10] Tian X Liu J Jet al L. Removal of inferior vena cava filter by open surgery after failure of endovenous retrieval. Front Cardiovasc Med. (2023) 10:1127886. 10.3389/fcvm.2023.1127886 37139130 PMC10150111

[11] Li X Haddadin I McLennan G Farivar B Staub D Beck A Inferior vena cava filter—comprehensive overview of current indications, techniques, complications and retrieval rates. Vasa. (2020) 49(6):449–62. 10.1024/0301-1526/a000887 32660360

[12] Desai KR Pandhi MB Seedial SM Errea MF Salem R Ryu RK Retrievable IVC filters: comprehensive review of device-related complications and advanced retrieval techniques. RadioFigureics. (2017) 37:1236–45. 10.1148/rg.2017160167 28696849

[13] Tian X Liu JL Li JY Liu X. Case report: endoluminal removal of a retrievable conical inferior vena cava filter with a ruptured retraction hook attached to the wall. Front Surg. (2022) 9:985060. 10.3389/fsurg.2022.985060 36439536 PMC9684660

[14] Nunnelee JD. Review of an article: oral rivaroxaban for symptomatic venous thromboembolism. The EINSTEIN investigators et al. N Engl J Med. (2010) 363(26):2499–510. 10.1056/NEJMoa1007903 21558032

[15] Murphy KD. Superior vena cava filters. Tech Vasc Interv Radiol. (2004) 7:105–9. 10.1053/j.tvir.2004.02.007 15252768

[16] Muriel A Jimenez D Aujesky D Bertoletti L Decousus H Laporte S Survival effects of inferior vena cava filter in patients with acute symptomatic venous thromboembolism and a significant bleeding risk. J Am Coll Cardiol. (2014) 63:1675–83. 10.1016/j.jacc.2014.01.058 24576432

[17] Milovanovic L Kennedy SA Midia M. Procedural and indwelling complications with inferior vena cava filters: frequency, etiology, and management. Semin Intervent Radiol. (2015) 32:34–41. 10.1055/s-0034-1396962 25762846 PMC4346045

[18] Zeng X Zhou J Zhou Q Huang Z. Case report: pericardial tamponade and hemothorax after superior vena cava filter removal. Front Cardiovas Med. (2022) 9:863732. 10.3389/fcvm.2022.863732 PMC9204137 35722085

[19] Kuyumcu G Walker TG. Inferior vena cava filter retrievals, standard and novel techniques. Cardiovasc Diagn Ther. (2016) 6(6):642–50. 10.21037/cdt.2016.09.07 28123984 PMC5220200

[20] Daye D Walker TG. Novel and advanced techniques for complex IVC filter retrieval. Curr Treat Options Cardiovasc Med. (2017) 19(4):28. 10.1007/s11936-017-0529-3 28332097

[21] Liu J Jiang P Tian X Jia W Huang N-L Zhan H Clinical outcomes of retrievable inferior vena cava filters for venous thromboembolic diseases. J Comp Eff Res. (2022) 11(6):437–49. 10.2217/cer-2021-0291 35199584

